# Swarm incursions of reassortants of highly pathogenic avian influenza virus strains H5N8 and H5N5, clade 2.3.4.4b, Germany, winter 2016/17

**DOI:** 10.1038/s41598-017-16936-8

**Published:** 2018-01-08

**Authors:** Anne Pohlmann, Elke Starick, Christian Grund, Dirk Höper, Günter Strebelow, Anja Globig, Christoph Staubach, Franz J. Conraths, Thomas C. Mettenleiter, Timm Harder, Martin Beer

**Affiliations:** 1 Institute of Diagnostic Virology, Friedrich-Loeffler-Institut, Südufer 10, 17493 Greifswald-Insel, Riems Germany; 2 Institute of Epidemiology, Friedrich-Loeffler-Institut, Südufer 10, 17493 Greifswald-Insel, Riems Germany; 3 Institute of Molecular Virology and Cell Biology, Friedrich-Loeffler-Institut, Südufer 10, 17493 Greifswald-Insel, Riems Germany

## Abstract

The outbreak of highly pathogenic avian influenza H5Nx viruses in winter 2016/2017 was the most severe HPAI epizootic ever reported in Germany. The H5N8 and H5N5 viruses detected in birds in Germany in 2016/2017 represent a reassortant swarm of at least five distinct genotypes, which carried closely related HA segments derived from clade 2.3.4.4b. The genotypes of these viruses and their spatio-temporal distribution indicated a unique situation with multiple independent entries of HPAIV into Germany.

## Introduction

Highly pathogenic avian influenza (HPAI) is a lethal disease of gallinaceous poultry, which can also cause severe losses in domestic waterfowl as well as in aquatic wild birds. HPAI viruses (HPAIV) of the goose/Guangdong (gs/GD) lineage of subtype H5N1 emerged in 1996 in Southern China, continued since then to spread among poultry, evolved into numerous phylogenetic clades^1^ and, by reassortment with various avian influenza viruses (AIV), fanned out to generate multiple geno- and subtypes. Some of these lineages contain viruses with enhanced zoonotic properties causing fatal human infections following spill-over transmission from infected poultry. HPAIV of clade 2.3.4.4, subtype H5N8, rooted in Far East Asia in 2013^2^, and H5N8 viruses designated as Group A within clade 2.3.4.4 were found responsible for outbreaks in wild birds and poultry in Central Asia, Russia and Central Europe in 2014/15^3–6^. Furthermore, strains of this clade were transferred to North America and, after forming novel reassortants with AIV of North-American lineages (HPAIV H5N2), caused dramatic losses in U.S. poultry farms^7^. Transcontinental spread of these strains has been linked to dissemination by migratory wild bird populations in a chain of sequential transmission events^8^.

In summer 2016, reassortants of H5N8 Group B viruses within clade 2.3.4.4 were found in dead wild aquatic birds at lakes known as migratory bird moulting and resting places located along the China-Mongolia and Russia-Mongolia borders^9–11^. Similar viruses caused death in captive birds in Indian zoos^12^ and commercial poultry in Iran later in 2016^13^, and were also found in migratory birds and poultry in Egypt^14^. In fall 2016, new reassortants of this strain appeared in a massive outbreak among wild birds in central Europe including Germany^15,16^ and subsequently spread all over Europe.

During the course of the outbreaks in winter 2016/2017 it became obvious that more and different reassortants of the original Russian-Chinese strain independently reached Germany and caused the most severe HPAIV epizootic in wild birds and poultry ever reported in Germany. Here, we present a synopsis of the incursion events and a detailed genetic and phylogenetic analysis of the causative viruses.

## Results

The 2016/17 HPAI H5 epizootic started in Germany in early November 2016 when large numbers of carcasses of diving duck species, in particular Tufted ducks (*Aythya fuligula*), were found almost simultaneously at Lake Plön in Northern Germany and Lake Constance in the South. In both events, H5N8 HPAIV were detected with high loads in tissues including liver and brain of the ducks. Since then, 1271 HPAIV H5- infected wild birds of at least 47 species were detected across Germany. The spectrum of affected wild bird species varied over time with a predominance of diving duck species (Tufted ducks [*Aythya fuligula*], Common pochards [*Aythya ferina*]) early in the epizootic which shifted towards gulls, birds of prey (including 18 white-tailed eagles [*Haliaeetus albicilla*]) and herons towards the end of the epizootic. In addition to wild birds, 92 poultry holdings and 15 zoos were affected. In most of the poultry cases, including ducks and geese, severe clinical signs and increased mortality was reported.

**Figure 1:**
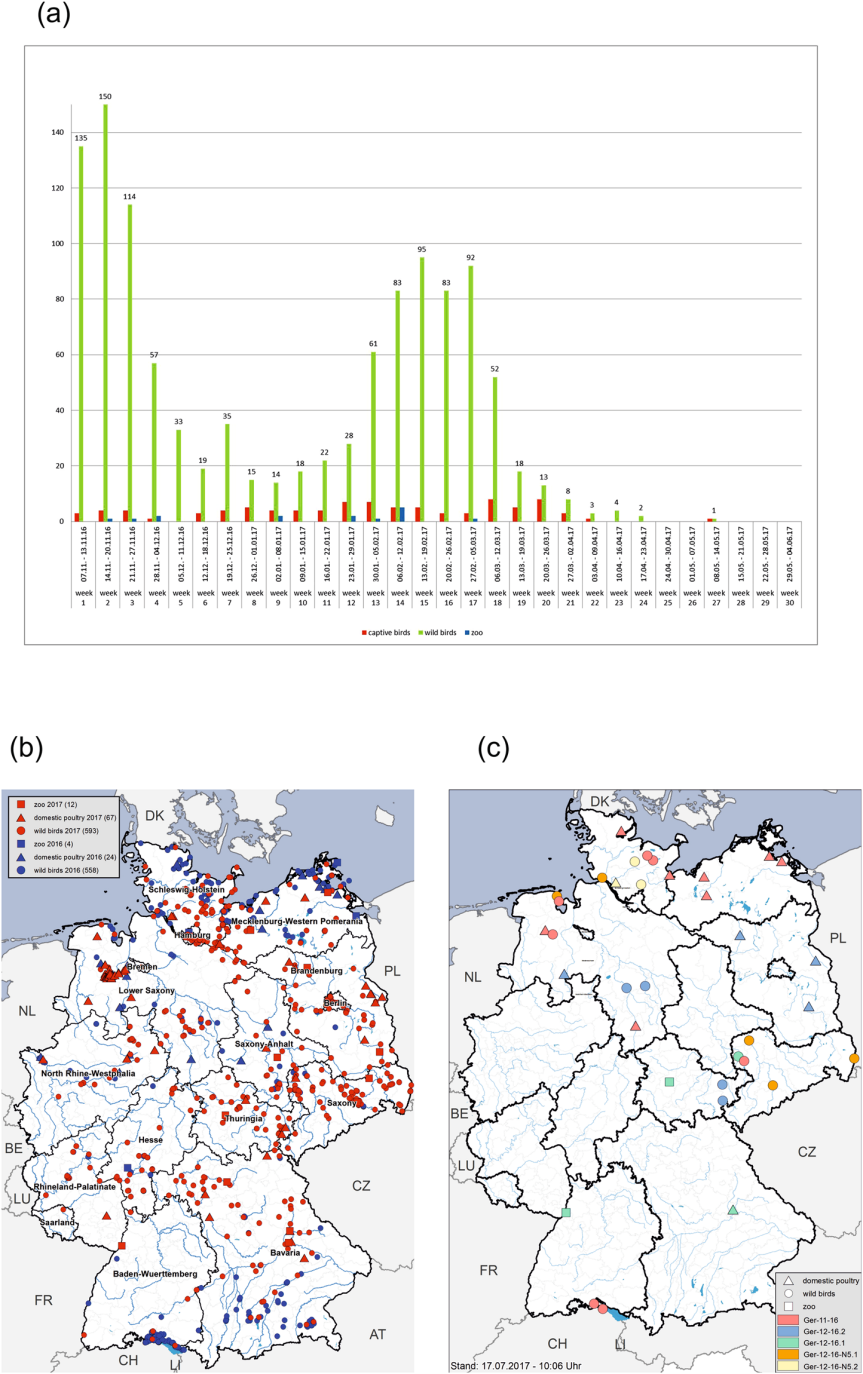
(**a**) Number of HPAI H5Nx cases in Germany 2016/2017 per week in wild birds (green), domestic poultry (red) and zoos (blue). (**b**) Geographical distribution of HPAI H5Nx cases in Germany 2016/2017 Distribution in time showing H5Nx cases in 2016 (blue) and 2017 (red). (**c**) Geographical distribution of distinct reassortants. Maps were plotted using ArcGIS Desktop software (version 10.3.1; ESRI, Redlands, CA, USA; http://www.esri.com/).

The temporal course of the outbreak in wild birds was characterized by at least two waves, with maxima in mid-November 2016 and mid-February 2017, respectively (Fig. 1a). Likewise, the geographical spread of the cases shows a distinct pattern with cases dominating the coastal regions of the North and Baltic Sea as well as inland lakes in the South in 2016, and Central Germany in 2017, respectively (Fig. 1b).

### Genetic analyses

**Figure 2:**
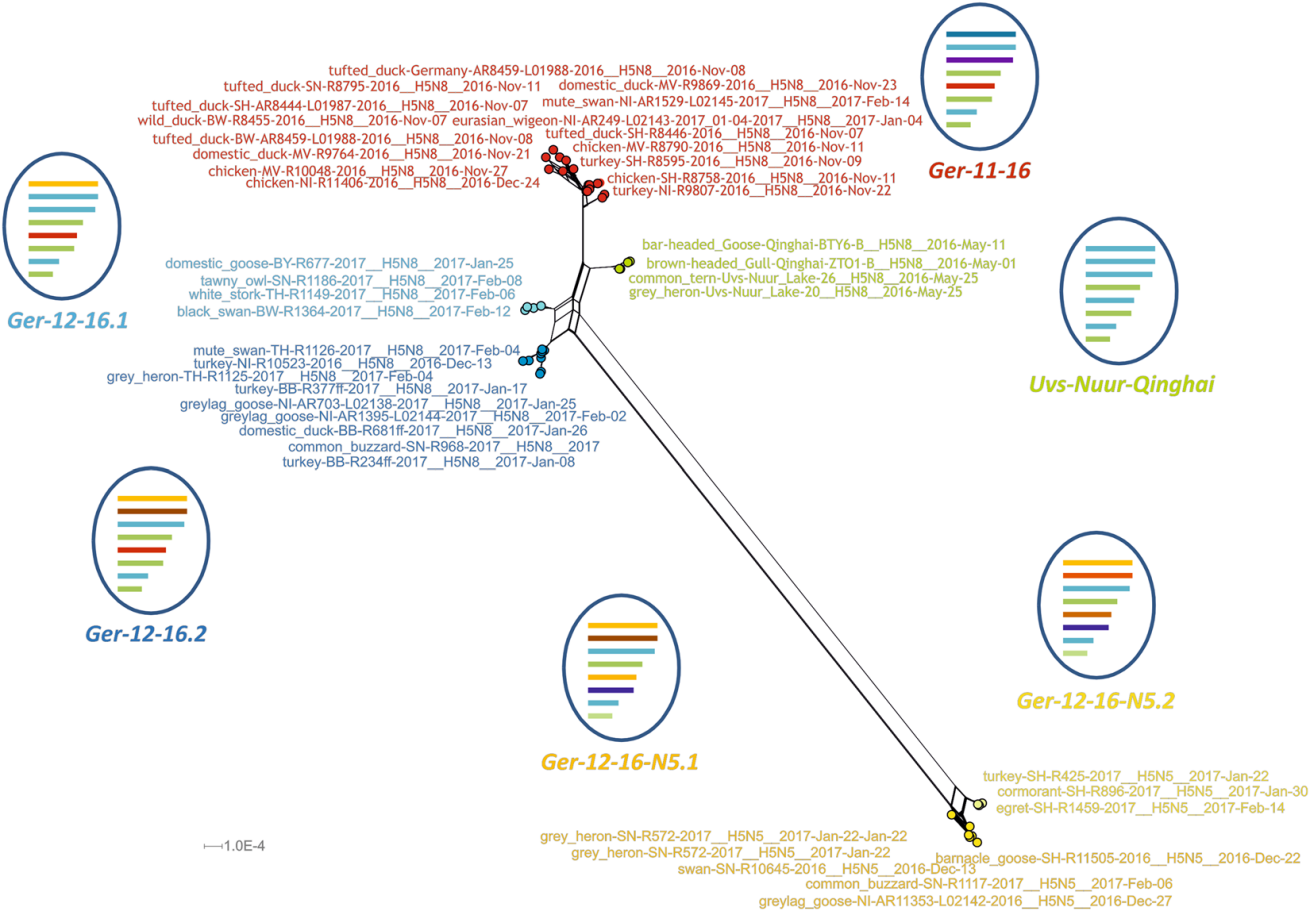
Super-network from Maximum Likelihood (ML) trees of PB2, PB1, PA, HA, NP, NA, MP, and NS genes of viruses from Germany 2016/2017 and selected reference strains. ML analyses were done using RAxML including bootstrapping for 1000 iterations. Network analysis was done with SplitsTree4. Schematic reassortant viruses grouped according to phylogenetic results are shown for each cluster.

**Figure 3:**
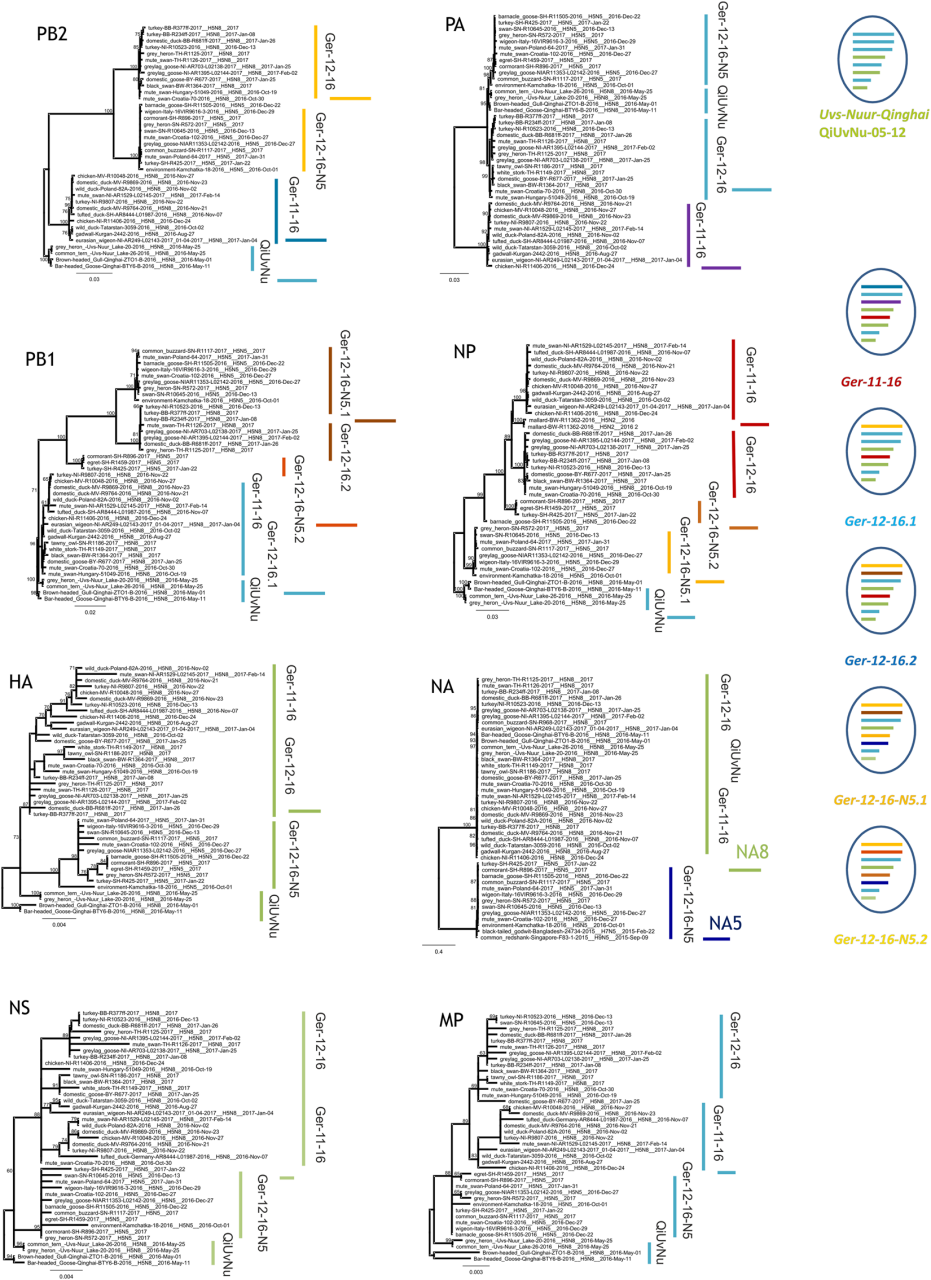
Phylogenetic analyses of PB2, PB1, PA, HA, NP, NA, MP, and NS genes from viruses Germany 2016/2017 and selected reference strains done by Maximum likelihood using RAxML. Bootstrap values of 1000 cycles are included. Scale bars indicate nucleotide substitutions per site. Reassortants grouped according to phylogenetic results are shown to the right.

For a comprehensive genetic analysis, a subset of 35 positive field samples from poultry and wild birds was sequenced yielding, in total, more than 240 genome segments for our analysis (Supplementary Table S1). The network analyses of these genome information revealed three overarching groups of reassortants of which two groups each harbored two different reassortants, respectively (Fig. 2). These overall five reassortants co-circulated in poultry and wild birds temporally and, with some exceptions, also geographically. Reassortant group 1 identified by genotype “Ger-11-16” was detected at the beginning of the incursion in November 2016 (Lake Plön and Lake Constance). Viruses of group 2, genotype “Ger-12-16” were collected from mid-December onwards. This group contained two different reassortants (Ger-12-16.1; Ger-12-16.2) which could be clearly distinguished by different PB1 segments pointing to a sequential reassortment event (Fig. 3). The third group comprises H5 viruses of genotype “Ger-12-16-N5” (Fig. 2) with N5 as a new NA-subtype. These novel HPAIV H5N5-viruses were detected in Germany in December 2016 for the first time and two different reassortants (Ger-12-16-N5.1, Ger-12-16-N5.2) were identified. Ger-12-16-N5.1 H5N5 viruses harbored segments with sequences similar to those found in an environmental sample collected October 2016 in Kamschatka. These H5N5 viruses contained an NA segment that rooted in H9N5 or H7N5 viruses found 2015 in wader birds in Singapore or Bangladesh, respectively (A/common redshank/Singapore/F83-1/2015, KU144675; A/black-tailed godwit/Bangladesh/24734/2015, KY635758).Three of the H5N5 viruses contain 6 segments of *“Ger-12-16-N5”* but novel PB1 and NP segments (Fig. 3). These PB1 and NP segments were not present in H5N5 viruses found in 2016/2017 in other European countries^16^ pointing to further reassortment events.

The HA of the three reassortant groups form a cluster of sequences that rooted in lineage 2.3.4.4b, A/breeder duck/Korea/Gochang1/2014 (H5N8)^6^. The latter strain also provided the NS segments of all viruses and the NA N8 segment of the H5N8 viruses. The NS1 protein of the German H5N8 viruses, however, revealed a C-terminal truncation of 13 amino acids compared to A/breeder duck/Korea/Gochang1/2014 (H5N8). In contrast, the German H5N5 viruses expressed a full-length NS1 protein with several amino acid exchanges (D2E, T129I, N205S in the NS1 and D2E, T48A in the NS2 proteins), which indicates that the H5N5 viruses represented independent incursions (Fig. 3).

The PB2, PB1 and PA segments of all three reassortant groups were clearly distinct (Fig. 3). PB2 and PB1 of genotype *“Ger-11-16”* were similar but not identical to those of H5N8 strains found at migratory bird resting places in Russia and China (10–12) and rooted in LPAI viruses from Asia. PB2 and PB1 segments of genotypes *“Ger-12-16-N5”* and “*Ger-12-16”* had different origins (Fig. 3). In contrast, in PA segments genotypes *“Ger-12-16-N5”* and *“Ger-12-16”* were very similar to the H5N8 strains found in Russia and China^9–11^, while genotype *“Ger-11-16”* had a different PA segment (Fig. 3)^15^. Origin of the NP segments confirmed the mosaic reassortment patterns showing a common origin of NP for genotypes *“Ger-11-16”* and *“Ger-12-16”* and a different origin for *“Ger-12-16-N5”* viruses.

## Conclusions

The *five* reassortants we identified during the 2016/17 outbreak of HPAIV H5N8 and H5N5 in Germany ordered in three distinct groups, which indicate independent incursions. The independence of the incursion of the different reassortants into Germany is supported by their time of detection and the geographical distribution patterns (Fig. 1c). Group “Ger-11-16” dominated the first wave of the outbreak in November and December 2016, and was found predominantly in the coastal regions of the North Sea and Baltic Sea or at lakes in Southern Germany, respectively, in association with local mass mortality events in diving duck species. Viruses of this group had been found in wild water birds in Russia in Tatarstan, Kurgan, and at Lake Chany close to Novosibirsk between August and October 2016, i.e. before their first detection in Europe^11^. Genotype *“Ger-12-16”* which included two reassortants was present in the center of Germany from mid-December 2016. Similar viruses have been found in Southern Europe from October 2016 onwards. The third group of H5N5 viruses, genotype *“Ger-12-16-N5”* that independently entered Germany, was found predominantly in Northern Germany, but was first identified in mid-December in Southern Germany. Since HPAIV H5N5 was also detected in the Czech Republic and in Poland, it is tempting to speculate that the H5N5 incursion originated from this geographical area.

## Methods

Swabs or organs were tested for influenza A virus by using reverse transcription quantitative PCR. A representative subset of positive samples was selected based on the rational to cover the spectrum of different host species (including poultry), the affected geographical regions, the full temporal extent of the outbreak. A smaller subset of these samples was selected for virus isolation. All sampling procedures were carried out in accordance with relevant guidelines and legal regulations. Handling of potentially infectious samples and materials was strictly confined to a BSL3 laboratory environment. Isolates were obtained after the first passage in embryonated eggs from specific pathogen free (SPF) chicken flocks using standard procedures. Sequencing was done by Sanger or Next Generation sequencing using isolates or field samples as previously described^5,17^. RNA of samples was extracted using Trizol LS (ThermoFisher Scientific, Waltham, USA) and QIAamp Viral RNA Mini Kit (Qiagen, Hilden, Germany). When using field samples influenza genome segments were amplified with influenza-specific primers using Invitrogen Superscript III One-Step RT-PCR with Platinum Taq (ThermoFisher Scientific, Waltham, USA). The RT-PCR amplicons were sequenced by Sanger or Next Gerneration sequencing. For Next generation sequencing of isolates, RNA was extracted and used as template for cDNA synthesis with cDNA Synthesis System REF 001,11,117,831 (Roche, Mannheim, Germany). Fragmentation of the cDNA or influenza RT-PCR amplicons, respectively, was done with Covaris M220 ultrasonicator (Covaris Ltd, Brighton, UK) applying a target size of 300 bp. The sonicated cDNA were used for library preparation using IonTorrent Ion Xpress Barcode Adapters and GeneRead DNA Library L Core Kit (Qiagen, Hilden, Germany). Size exclusion of the library was done with Ampure XP magnetic beads (Beckman Coulter, Fullerton, USA). The libraries were quality checked using High Sensitivity DNA Chips and reagents on a Bioanalyzer 2100 (Agilent Technologies, Böblingen, Germany), quantized via qPCR with Kapa Library Quantification Kit Ion Torrent (Roche, Mannheim, Germany) and sequenced on an IonTorrent PGM (Thermo Scientific, Waltham, MA USA). Raw sequence data were quality-trimmed and screened for adapter and primer contamination. Consensus sequences were generated using an iterative assembly and mapping approach done with Newbler Genome Sequencer software (v.3.0; Roche, Mannheim Germany) and Geneious software suite (v.10.0.9; Biomatters, Auckland, New Zealand). Phylogenetic analyses of sequences were done using RAxML^18^, and SplitsTree4^19^ was used for network analyses. Sequences were deposited into the GISAID EpiFlu™ database. A detailed list and acknowledgement of the sequences retrieved from EpiFlu™ database is given in Supplementary Table S2.

## Electronic supplementary material

Table S1

Table S2

## References

[1] Smith GJ, Donis RO, World Health Organization/WorldOrganisation for Animal, H. F. & Agriculture Organization, H. E. W. G (2015). Nomenclature updates resulting from the evolution of avian influenza A(H5) virus clades 2.1.3.2a, 2.2.1, and 2.3.4 during 2013-2014. Influenza Other Respir Viruses.

[2] Zhong L (2014). The antigenic drift molecular basis of the H5N1 influenza viruses in a novel branch of clade 2.3.4. Vet Microbiol.

[3] Wu H (2014). Novel reassortant influenza A(H5N8) viruses in domestic ducks, eastern China. Emerg Infect Dis.

[4] Verhagen, J. H. *et al*. Wild bird surveillance around outbreaks of highly pathogenic avian influenza A(H5N8) virus in the Netherlands, 2014, within the context of global flyways. *Euro Surveill* **20** (2015). 10.2807/1560-7917.es2015.20.12.21069 25846491

[5] Harder T (2015). Influenza A(H5N8) Virus Similar to Strain in Korea Causing Highly Pathogenic Avian Influenza in Germany. Emerg Infect Dis.

[6] Lee YJ (2014). Novel reassortant influenza A(H5N8) viruses, South Korea, 2014. Emerg Infect Dis.

[7] Lee DH (2015). Intercontinental Spread of Asian-Origin H5N8 to North America through Beringia by Migratory Birds. J Virol.

[8] Global-Consortium-for-H5N8-and-Related-Influenza-Viruses (2016). Role for migratory wild birds in the global spread of avian influenza H5N8. Science.

[9] Lee, D. H. *et al*. Novel Reassortant Clade 2.3.4.4 Avian Influenza A(H5N8) Virus in Wild Aquatic Birds, Russia, 2016. *Emerg Infect Dis* **23**, 10.3201/eid2302.161252 (2017). 10.3201/eid2302.161252 PMC5324796 27875109

[10] Li M (2017). Highly Pathogenic Avian Influenza A(H5N8) Virus in Wild Migratory Birds, Qinghai Lake, China. Emerg Infect Dis.

[11] Marchenko VY (2017). Reintroduction of highly pathogenic avian influenza A/H5N8 virus of clade 2.3.4.4. in Russia. Arch Virol.

[12] Nagarajan S (2017). Novel Reassortant Highly Pathogenic Avian Influenza (H5N8) Virus in Zoos, India. Emerg Infect Dis.

[13] Ghafouri SA (2017). Clade 2.3.4.4 avian influenza A (H5N8) outbreak in commercial poultry, Iran, 2016: the first report and update data. Trop Anim Health Prod.

[14] Selim AA (2017). Highly Pathogenic Avian Influenza Virus (H5N8) Clade 2.3.4.4 Infection in Migratory Birds, Egypt. Emerg Infect Dis.

[15] Pohlmann A (2017). Outbreaks among Wild Birds and Domestic Poultry Caused by Reassorted Influenza A(H5N8) Clade 2.3.4.4 Viruses, Germany, 2016. Emerg Infect Dis.

[16] Fusaro, A. *et al*. Genetic Diversity of Highly Pathogenic Avian Influenza A(H5N8/H5N5) Viruses in Italy, 2016–17. *Emerg Infect Dis* **23**, 10.3201/eid2309.170539 (2017). 10.3201/eid2309.170539 PMC5572881 28661831

[17] Juozapaitis M (2014). An infectious bat-derived chimeric influenza virus harbouring the entry machinery of an influenza A virus. Nat Commun.

[18] Stamatakis A (2014). RAxML version 8: a tool for phylogenetic analysis and post-analysis of large phylogenies. Bioinformatics.

[19] Huson DH, Bryant D (2006). Application of phylogenetic networks in evolutionary studies. Mol Biol Evol.

