# Supplementary material for: Swarm incursions of reassortants of highly pathogenic avian influenza virus strains H5N8 and H5N5, clade 2.3.4.4b, Germany, winter 2016/17
Source: Sci Rep. 2018 Jan 8;8:15. doi: 10.1038/s41598-017-16936-8 (PMC5758748; doi:10.1038/s41598-017-16936-8)
Supplement: Supplementary file 1 — Table S1 [file 41598_2017_16936_MOESM1_ESM.doc]

**Swarm incursions of reassortants of highly pathogenic avian influenza virus strains H5N8 and H5N5, clade 2.3.4.4b, Germany, winter 2016/17**

Anne Pohlmann^1^, Elke Starick^1^, Christian Grund^1^, Dirk Höper^1^, Günter Strebelow^1^, Anja Globig^2^, Christoph Staubach^2^, Franz J. Conraths^2^, Thomas C. Mettenleiter^3^, Timm Harder^1^*, Martin Beer^1^

^1^ Institute of Diagnostic Virology, Friedrich-Loeffler-Institut, Südufer 10, 17493 Greifswald-Insel Riems, Germany

^2^ Institute of Epidemiology, Friedrich-Loeffler-Institut, Südufer 10, 17493 Greifswald-Insel Riems, Germany

^3^ Institute of Molecular Virology and Cell Biology, Friedrich-Loeffler-Institut, Südufer 10, 17493 Greifswald-Insel Riems, Germany

***Corresponding author:**

Prof. Dr. Timm Harder; phone: 004938351-7-1546; fax: 004938351-7-1226; e-mail: timm.harder@fli.de

**Keywords**: HPAI, Avian Influenza, H5N8, clade 2.3.4.4, epizootic, reassortants

| Reassortante designation | Accession Number | Designation | Host | German Federal State | Collection date |
| --- | --- | --- | --- | --- | --- |
| Ger-11-16 | EPI_ISL_262055 | eurasian_wigeon-NI-AR249-L02143__H5N8 | Anas penelope | Niedersachsen | 04.01.2017 |
| Ger-11-16 | EPI_ISL_249692 | chicken-NI-R11406__H5N8 | Gallus gallus | Niedersachsen | 24.12.2016 |
| Ger-11-16 | EPI_ISL_237945 | tufted_duck-Germany-AR8459-L01988__H5N8 | Aythya fuligula | Baden-Württemberg | 08.11.2016 |
| Ger-11-16 | EPI_ISL_238037 | turkey-SH-R8595__H5N8 | Meleagris gallopavo | Schleswig-Holstein | 09.11.2016 |
| Ger-11-16 | EPI_ISL_237944 | tufted_duck-Germany-AR8444-L01987__H5N8 | Meleagris gallopavo | Schleswig-Holstein | 07.11.2016 |
| Ger-11-16 | EPI_ISL_262057 | mute_swan-NI-AR1529-L02145__H5N8 | Cygnus olor | Niedersachsen | 14.02.2017 |
| Ger-11-16 | EPI_ISL_256818 | domestic_duck-MV-R9764__H5N8 | Domestic duck | Mecklenburg-Vorpommern | 21.11.2017 |
| Ger-11-16 | EPI_ISL_256817 | turkey-NI-R9807__H5N8 | Meleagris gallopavo | Niedersachsen | 22.11.2017 |
| Ger-11-16 | EPI_ISL_238039 | chicken-SH-R8758__H5N8 | Gallus gallus | Schleswig-Holstein | 11.11.2016 |
| Ger-11-16 | EPI_ISL_238038 | chicken-MV-R8790__H5N8 | Gallus gallus | Mecklenburg-Vorpommern | 11.11.2016 |
| Ger-11-16 | EPI_ISL_249691 | chicken-MV-R10048__H5N8 | Gallus gallus | Mecklenburg-Vorpommern | 27.11.2016 |
| Ger-11-16 | EPI_ISL_241249 | domestic_duck-MV-R9869__H5N8 | Domestic duck | Mecklenburg-Vorpommern | 23.11.2016 |
| Ger-11-16 | EPI_ISL_237732 | tufted_duck-SH-R8446-2016__H5N8 | Aythya fuligula | Schleswig-Holstein | 07.11.2016 |
| Ger-11-16 | EPI_ISL_237792 | wild_duck-BW-R8455-2016__H5N8 | Wild duck | Baden-Württemberg | 07.11.2016 |
| Ger-11-16 | EPI_ISL_238040 | tufted_duck-SN-R8795-2016__H5N8 | Aythya fuligula | Sachsen | 11.11.2016 |
| Ger-12-16.1 | EPI_ISL_249682 | goose-BY-R677__H5N8 | Domestic goose | Bayern | 25.01.2017 |
| Ger-12-16.1 | EPI_ISL_258526 | white_stork-TH-R1149-2017__H5N8 | Ciconia ciconia | Thüringen | 06.02.2017 |
| Ger-12-16.1 | EPI_ISL_258527 | tawny_owl-SN-R1186-2017__H5N8 | Strix aluco | Sachsen | 08.02.2017 |
| Ger-12-16.1 | EPI_ISL_261687 | black_swan-BW-R1364-2017__H5N8 | Cygnus atratus | Baden-Württemberg | 12.02.2017 |
| Ger-12-16.2 | EPI_ISL_262058 | greylag_goose-Germany-AR1395-L02144__H5N8 | Anser anser | Niedersachsen | 02.02.2017 |
| Ger-12-16.2 | EPI_ISL_262059 | greylag_goose-NI-AR703-L02138__H5N8 | Anser anser | Niedersachsen | 25.01.2017 |
| Ger-12-16.2 | EPI_ISL_247429 | domestic_duck-BB-R681ff__H5N8 | Domestic duck | Brandenburg | 26.01.2017 |
| Ger-12-16.2 | EPI_ISL_249681 | turkey-BB-R234ff__H5N8 | Meleagris gallopavo | Brandenburg | 09.01.2017 |
| Ger-12-16.2 | EPI_ISL_240892 | turkey-NI-R10523__H5N8 | Meleagris gallopavo | Niedersachsen | 13.12.2016 |
| Ger-12-16.2 | EPI_ISL_257731 | turkey-BB-R377ff__H5N8 | Meleagris gallopavo | Brandenburg | 17.01.2017 |
| Ger-12-16.2 | EPI_ISL_258415 | grey_heron-TH-R1125-2017__H5N8 | Ardea cinerea | Thüringen | 04.02.2017 |
| Ger-12-16.2 | EPI_ISL_258661 | mute_swan-TH-R1126-2017__H5N8 | Cygnus olor | Thüringen | 04.02.2017 |
| Ger-12-16-N5.1 | EPI_ISL_243050 | barnacle_goose-SH-R11505__H5N5 | Branta leucopsis | Schleswig-Holstein | 22.12.2016 |
| Ger-12-16-N5.1 | EPI_ISL_240893 | swan-SN-R10645__H5N5 | Cygnus | Sachsen | 13.12.2016 |
| Ger-12-16-N5.1 | EPI_ISL_262056 | greylag_goose-NI-AR11353-L02142__H5N5 | Anser anser | Niedersachsen | 27.12.2016 |
| Ger-12-16-N5.1 | EPI_ISL_260058 | grey_heron-SN-R572-2017__H5N5 | Ardea cinerea | Sachsen | 22.01.2017 |
| Ger-12-16-N5.1 | EPI_ISL_259074 | common_buzzard-SN-R1117-2017__H5N5 | Buteo buteo | Sachsen | 06.02.2017 |
| Ger-12-16-N5.2 | EPI_ISL_243049 | turkey-SH-R425__H5N5 | Meleagris gallopavo | Schleswig-Holstein | 22.01.2017 |
| Ger-12-16-N5.2 | EPI_ISL_260059 | cormorant-SH-R896__H5N5 | Phalacrocorax | Schleswig-Holstein | 30.01.2017 |
| Ger-12-16-N5.2 | EPI_ISL_259525 | egret-SH-R1459__H5N5 | Egret | Schleswig-Holstein | 14.02.2017 |

**Supplementary Table S1. Overview of viruses, reassortant designations, and relevant metadata.**
